# Variability in the Carbon Storage of Seagrass Habitats and Its Implications for Global Estimates of Blue Carbon Ecosystem Service

**DOI:** 10.1371/journal.pone.0073748

**Published:** 2013-09-05

**Authors:** Paul S. Lavery, Miguel-Ángel Mateo, Oscar Serrano, Mohammad Rozaimi

**Affiliations:** 1 School of Natural Sciences and Centre for Marine Ecosystems Research, Edith Cowan University, Joondalup, WA, Australia; 2 Centro de Estudios Avanzados de Blanes, Consejo Superior de Investigaciones Cientificas, Blanes, Girona, Spain; 3 The UWA Oceans Institute, The University of Western Australia, Crawley, WA, Australia; 4 School of Environmental and Natural Resource Sciences, Universiti Kebangsaan Malaysia, Bangi, Selangor, Malaysia; Dauphin Island Sea Lab, United States of America

## Abstract

The recent focus on carbon trading has intensified interest in ‘Blue Carbon’–carbon sequestered by coastal vegetated ecosystems, particularly seagrasses. Most information on seagrass carbon storage is derived from studies of a single species, *Posidonia oceanica*, from the Mediterranean Sea. We surveyed 17 Australian seagrass habitats to assess the variability in their sedimentary organic carbon (C_org_) stocks. The habitats encompassed 10 species, in mono-specific or mixed meadows, depositional to exposed habitats and temperate to tropical habitats. There was an 18-fold difference in the C_org_ stock (1.09–20.14 mg C_org_ cm^−3^ for a temperate *Posidonia sinuosa* and a temperate, estuarine *P. australis* meadow, respectively). Integrated over the top 25 cm of sediment, this equated to an areal stock of 262–4833 g C_org_ m^−2^. For some species, there was an effect of water depth on the C_org_ stocks, with greater stocks in deeper sites; no differences were found among sub-tidal and inter-tidal habitats. The estimated carbon storage in Australian seagrass ecosystems, taking into account inter-habitat variability, was 155 Mt. At a 2014–15 fixed carbon price of A$25.40 t^−1^ and an estimated market price of $35 t^−1^ in 2020, the C_org_ stock in the top 25 cm of seagrass habitats has a potential value of $AUD 3.9–5.4 bill. The estimates of annual C_org_ accumulation by Australian seagrasses ranged from 0.093 to 6.15 Mt, with a most probable estimate of 0.93 Mt y^−1^ (10.1 t. km^−2^ y^−1^). These estimates, while large, were one-third of those that would be calculated if inter-habitat variability in carbon stocks were not taken into account. We conclude that there is an urgent need for more information on the variability in seagrass carbon stock and accumulation rates, and the factors driving this variability, in order to improve global estimates of seagrass Blue Carbon storage.

## Introduction

There is considerable interest in quantifying the capacity of the World’s ecosystems to trap and sequester carbon (C). The recent focus on C trading and C pricing has intensified this interest, since net C storage provides one means of valuing these ecosystems. In the early 1980s, [1] highlighted the importance of vegetated coastal habitats as carbon sinks, though since then most work on the ability of ecosystems to capture C has focused on terrestrial ecosystems. However, the publication of the ‘Blue Carbon’ report [2] has highlighted the potential of coastal marine ecosystems to sequester carbon. For example, they estimate that coastal marine systems capture up to half of the CO_2_ emissions from the World’s transport sector. These ecosystems include mangroves, coral reefs, saltmarshes and seagrasses, with seagrasses having a disproportionately large C storage potential relative to their global area [3].

From necessity, there has been a tendency to generalise the C_org_-capture attributes of seagrasses from a very limited data set, with almost all of the estimates being based largely on the C_org_ content of sediments from Mediterranean *Posidonia oceanica* meadows (e.g. [2], [4]. However, *P. oceanica* is unusual in its ability to capture C. Its vertical growth dynamics and recalcitrant tissues produce a deep mat of plant debris that can extend many meters down into the sediment and persist for millennia, resulting in massive C storage, up to 40–410 kg C_org_ m^−2^ [5]–[7]. As far as is known, no other seagrass has this attribute and, in a functional form model of seagrasses, Walker et al. [8] placed *P. oceanica* as an outlier. Nelleman et al. [2] recognized that their assumptions were likely to have produced an upper estimate of the Blue Carbon sink, in part because of uncertainties in the C burial rates of different seagrass ecosystems.

The other 70-plus species of seagrasses [9] have a wide variation in traits relevant to C capture and storage. This includes differences in their rates of primary production, their below ground biomass, the recalcitrance of the C_org_ in their organs, the ability of their canopies to trap allochthonous carbon and the conditions in their sediments that drive C_org_ preservation (see review of factors in [10]). For most of the World’s seagrass species, and for most of their global distribution, there is little knowledge of their carbon stocks or regional cover [11], though for a wide range of seagrass species an absolute C flux to the refractory compartment was estimated ranging from 1.8 to 177.8 g C m^−2^ y^−1^ (median: 56.2 g C m^−2^ y^−1^), representing from around 5% to 65% of total plant production (median:18% [10]). Also, a review of 219 seagrass sediment data sets, encompassing 20 species, showed significant variation in organic matter content, with C_org_ ranging from 0.1 wt % to 11.0 wt % [12]. Recently, a first attempt to compile global C_org_ data [13] examined 946 distinct seagrass meadows across the globe. They estimated an average C_org_ in the top meter of seagrass soils at 2.5 wt % (median 1.8 wt %). Using the rough latest estimates of total area of the Earth covered by seagrass meadows (between 300,000 and 600,000 km^2^), they come to a conservative estimate of a global stock from 4.2 to 8.4 Pg C_org_ for the top meter. A preliminary regional breakdown of the areal stock is also provided showing the highest areal stocks in the Mediterranean (372.4 Mg C_org_ ha^−1^ ± 74.5, n = 29; [13]) but no details on habitat or species stock distribution can be given because of data set limitations.

In addition to the variability among seagrass species, the range of habitats in which they occur is also likely to affect their C storage potential. Seagrasses occur across a range of depositional environments, from estuaries to exposed coastal environments [14]. They occur at a range of water depths which influences their net C balances [15], [16] and the organic C preservation due to differences in sediment grain-size [17]. They also occur across latitudinal ranges and habitats with significant temperature variation that can affect sediment respiration and remineralisation rates [18]. Consequently, there are likely to be species-habitat interactions that will strongly influence the capture and retention of sedimentary C_org_.

This paper presents the results of an initial survey of several species of Australian seagrasses to assess the variability in their sedimentary C stocks. While not fully comprehensive in the diversity of species examined it does, nonetheless, include about one-third of the Australian seagrass species, with estimates of C_org_ accumulation rates, and provides an initial contribution to broadening our understanding of the variability in the sedimentary C_org_ stocks of seagrass habitats. We also set out to test whether the variability in sedimentary C_org_ stocks among seagrasses was sufficient to warrant its inclusion in regional and global estimates of seagrass C_org_ storage, or whether a single global seagrass average (such as that currently based largely on *P. oceanica*) produces similar estimates. While our study focuses on Australian seagrasses, Australia is in a unique position of having some of the World’s largest and most diverse seagrass resources over a wide range of climates and habitat types, and encompasses much of the kind of variability found in seagrass ecosystems globally.

## Methods and Materials

### Ethics Statement

This research was approved by the Edith Cowan University Ethics Committee following submission of an ethics declaration. The collection of seagrass and sediment core samples undertaken for this research were approved through the issuing of collection permits by the Department of Conservation and Environment in Western Australia and the Department of Primary Industries in Queensland.

### Sedimentary Carbon Characteristics

Sediment cores were extracted from 17 mono-specific or mixed-species meadows of seagrass (Table 1). The meadows sampled incorporated tropical, sub-tropical and temperate climates as well as inter-tidal and sub-tidal habitats. The sampling design was not orthogonal as not all species occurred in all habitat types; thus, some species were sampled in only one location, while others were sampled in both inter- and sub-tidal habitats or in sub-tidal habitats of different depths.

**Table 1:** Location of seagrass meadow sampling sites.

Species	Zone	Location	Habitat	S	E
*Amphibolis antarctica*	subtropical	Shark Bay, WA	Sub-tidal	7144460	772637
			Inter-tidal	7144289	772989
	temperate	Geographe Bay, WA	Sub-tidal5m	6280864	353582
			Sub-tidal 10m	6282348	342501
*C. rotundata /H.uninervis*	tropical	Green Is., QLD	Sub-tidal	8147054	390456
*C. rotundata/S. isoetifolium*	tropical	Green Is., QLD	Sub-tidal	8147003	390468
*Cymodocea serrulata*	tropical	Trinity Inlet, QLD	Sub-tidal	8132670	369620
*Halodule uninervis*	tropical	Trinity Inlet, QLD	Sub-tidal	8131418	373498
			Inter-tidal	8131418	373510
*Halophila ovalis*	tropical	Trinity Inlet, QLD	Sub-tidal	8131053	371447
*Posidonia australis*	subtropical	Shark Bay, WA	Sub-tidal	7144460	772637
			Inter-tidal	7144289	772989
	temperate	Waychinicup Inlet	Sub-tidal	6137832	621812
*Posidonia sinuosa*	temperate	Geographe Bay, WA	Sub-tidal5m	6275712	336571
			Sub-tidal 10m	6277434	336006
*T. hemprichii/C. rotundata*	tropical	Green Is., QLD	Sub-tidal	8146599	390891
*Zostera muelleri*	tropical	Trinity Inlet, QLD	Sub-tidal	8131308	373498

Location of seagrass meadow sampling sites. The locations are given in UTM using WSG84 map datum and are central points of the study sites. *C. rotundata* = *Cymodocea rotundata*; *T. hemprichii* = *Thalassia hemprichii*.

At all sites except the *Posidonia australis* meadow at Waychinicup Inlet, PVC cores (i.d. 47 mm) were manually inserted into the sediments to a depth of 30 cm at three randomly located positions. The cores had serrated bottom edges to assist in cutting through seagrass rhizomes and were gently turned while being pushed or hammered into the sediments. The cores were retrieved, capped and returned to the boat where they were stacked vertically in a cool box until returning to the laboratory. At Waychinicup Inlet, PVC cores were inserted by manual hammering to the maximum possible depth (> 2.5 m). Sample compaction during coring was less than 25% in all cases.

In the laboratory, the sediments were extruded by inserting a plunger at the bottom of the cores and carefully drawing the PVC liner down over the plunger. The cores were sliced into 3 cm sections, at 0–3, 6–9, 12–15, 18–21 and 24–27 cm intervals. The slices were split into two sub-samples, with one sub-sampled retained for organic carbon analysis and the other for organic matter (Loss on Ignition, or LOI) and carbonate analyses. Cores from Waychinicup Inlet were sliced every cm for the first 30 cm. Analyses were performed on 1 cm sections corresponding to the depth ranges analysed for the other cores (e.g. usually the 2 cm section to correspond with the 0–3 cm section of the other cores).

### Organic Content and Carbonate Content

Each sub-sample was weighed before and after drying at 50°C for 48 h to determine bulk density and porosity. The samples were then ground in a ball mill and combusted at 450°C for 4 h to determine LOI [19] and then for 2 h at 950°C to determine the carbonate content [20]. All combustions included reference samples of pure glucose and calcium carbonate to correct for incomplete combustion of C_org_ and carbonates.

### Organic Carbon Content

The sub-sample for organic carbon analysis was dried, weighed and then dry-sieved through a 1 mm mesh to remove coarse inorganic particles. The remaining samples were then acidified with 4% HCl to remove inorganic carbon, washed in deionised water then centrifuged (3400 revolutions per minute, for 5 minutes) and the supernatant with acid residues carefully removed by pipette, avoiding resuspension. The residual samples were re-dried and then capsulated for %C and δ^13^C analyses using an ANCA-NT 20–20 Stable Isotope Analyser connected to an ANCA-NT Solid/Liquid Preparation Module (PDZ Europa instruments). δ^13^C values were reported relative to v-PDB standard. Percentage C was calculated for the bulk (pre-sieved and pre-acidified) samples.

### Estimating Australian Seagrass C_org_ Stocks and Accumulation Rates and the Effect of Including Inter-habitat Variability on Estimates

To examine the effect of incorporating the natural variability in sedimentary organic carbon storage of seagrasses into regional estimates of seagrass C_org_ stocks and accumulation rate, we estimated the total sedimentary C_org_ stock (C_stock_) of the top 25 cm of seagrass habitat in Australia as:
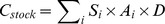
where, *i* refers to the 6 regions of Australia for which seagrass areas have been reported (Table 2), S_i_ is the mean C_org_ stock of the seagrasses representative of each region and measured in this study expressed in mg m^−3^, A_i_ is the estimated area of seagrass in each region expressed in m^2^) (Table 2), and D is the depth of sediment layer in m (in this case, 0.25 m). The stock was integrated over 25 cm as our deepest section of sediment sampled bracketed the 24–27 cm range, and 25 cm is convenient for normalization to the top 1 m of sediment, which has been examined in other studies (e.g. Fourqurean et al. 2012). The seagrass species considered representative of each region was based by matching [22] assessment of the dominant species in each region (Table 2) with the most morphologically similar species for which we had measured C_org_ stocks (Table 1). Where more than one species was likely to contribute significantly to the total area of seagrass, we weighted the contribution to the C stock equally among all the species.

**Table 2:** Estimates of seagrass area in various region of Australia.

State	Area(km^2^)	Habitat	Pre-dominantspecies	Source
New SouthWales	15	Estuarine	*P, Z, H*	1
	154	Estuarine		^2^
	**161**	Estuarine		^3^
Tasmania	60	Embayments	*P, Aa, Z, H*	1
	111	NW coast		^4^
	**845**	Varied		^5^
Victoria	10	Embayments	*P, A, Z, H*	1
	**470**	Estuarine		^5^
SouthAustralia	>5230	Varied	*P, A, Z*	1
	**9620**	Varied		^6^
QLD/NT/TS	2320	Embayments	*H, Hd, C* + smallareas of	1,7, 5, 8
	6000	Embayments	*Th, E and Tc*	
	**56473**	Varied		
WesternAustralia	2200	Varied	*P, A* in the SW;≥26 species	1
	**25000**	Varied	in NW, incl.*H, Hd* & *C*	
**Total**	**92569**			

Estimates of seagrass area in various region of Australia. Bold indicate the estimates which were used in the calculations of national seagrass C_org_ stocks and accumulation rates.

P = *Posidonia* spp.;

Z = *Zostera* spp.;

H = *Halophila* spp.;

A = *Amphibolis* spp;

Aa = *Amphibolis antarctica*;

Hd = *Halodule* spp.;

C = *Cymodocea* spp.;

Th = *Thalassia hemprichii*;

E = *Enhalus acroides*;

Tc = *Thalassodendron ciliatum*.

Sources:

1 [22]; ^2^ [48]; ^3^ [49]; ^4^ [50]; ^5^ [51]; ^6^ [52]; ^7^ [53]; ^8^ [54]cited [55].

For accumulation rates, a similar approach was taken to produce a range of possible accumulation rates and to assess the effect of incorporating inter-habitat and inter-species variability in the estimates. The organic carbon accumulation rate C_accum_ (t C_org_ y^−1^) was determined as:
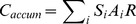
where, *i* refers to the 6 regions for which seagrass areas have been reported (Table 2), S_i_ is the mean organic carbon stock (mg m^−3^) in the top 25 cm of the seagrasses representative of each region and that were measured in this study, A_i_ is the estimated area (m^2^) of seagrass in each region (Table 2) and R is the rate of sediment accumulation (m y^−1^). The stocks were the mean of all depth layers, which better represents the medium-to long-term accumulation of C than considering only the top layer.

The rate of C accumulation is highly dependent on the rate of sediment accumulation. In the absence of dating for each of the cores, we assumed a range of sedimentation rates based on published literature and unpublished dating results that we have recently obtained for other seagrass areas throughout Australia (Table 3). The published rates show a large range of sediment accumulation rates from as low as 0.15 mm y^−1^ to 9.9 mm y^−1^ in seagrass habitats and over 17 mm y^−1^ in tropical lagoon habitats. Therefore, we applied a range of accumulation rates. We ignored the highest reported rate as this applied to tropical lagoon environments with no evidence that these supported seagrasses. Within the remaining range (0.15 to 9.9 mm y^−1^) are data derived from seagrass core dating studies. The maximum sediment accumulation rate of 9.9 mm y^−1^ [23] was based on Pb-210 dating at one site and relates to the past 60 years. For a nearby meadow they estimated an accumulation rate of 4.7 mm y^−1^ for the past 60 years and for the Holocene an average of 0.44 mm y^−1^ (based on ^14^C dating). This range illustrate the wide range of accumulation rates when considering short- vs long-term periods, reflecting a combination of factors such as human impacts on sedimentation dynamics as well as diagentic process (i.e. organic matter decomposition and sediment compaction with ageing). We have a number of dated *Posidonia australis* sediment core profiles from Oyster Harbour, Western Australia that indicate sediment accumulation rates in the order of 1.45–2.43 mm y^−1^ over the past 70–80 years (unpublished data). Together, these data indicated that sediment accumulation rates in seagrass meadows are likely to be in the range of 0.15–9.9 mm y^−1^ but C and lead dating suggesting accumulation rates for *Posidonia* species in the order of 1–1.5 mm y^−1^ in recent times. To capture this uncertainty, we used three representative sediment accumulation rates in seagrass meadows (0.15, 1.5 and 9.9 mm y^−1^) to calculate C_org_ accumulation rates:.

**Table 3:** Published sediment accumulation rates (by depth) for Australian coastal marine ecosystems and *P. oceanica* from the Mediterranean Sea.

Site	Habitat	Sedimentationrate (mm y^−1^)	Reference
Morton Bay	Inter-tidal	1.3–2.7	[56]
Ningaloo Reef	Fringingreef	1.46–9.88	[57]
SE Australia	Depositional	14.2–17.3	[58]
Fitzroy R., QLD	Estuary	15	[59]
Herbert R., QLD	Depositionalbay	1.11–11.4	[60]
GBR Nara Inlet	Inlet	1	[61]
Sydney	Nearshoreshelf	2–4	[62]
Port Phillip Bay	Embayment	1.5	[63]
Far North QLD	Inner shelf	0.4–1	[64]
Herbert R., QLD,	Tidalmud flats	0.3–8.5	[65]
Spencer Gulf	Seagrass	0.15–0.25	[66]
Sydney, Botany Bay	Seagrass	4.7–9.9	[23]
Mediterranean Sea	Seagrass	0.61–4.1	[5]
Mediterranean Sea	Seagrass	1	[42]
Albany, WA	Seagrass	1.45–2.43	Unpublisheddata
**Total Range**		**0.15–17.3**	
**Seagrass range**		**0.15–9.9**	

NB: Mass Accumulation Rates (MAR) were not used unless sediment bulk density data were available to convert the MAR to depth accumulation rates.

## Results

### Seagrass Sediment Characteristics

**Figure 1:**
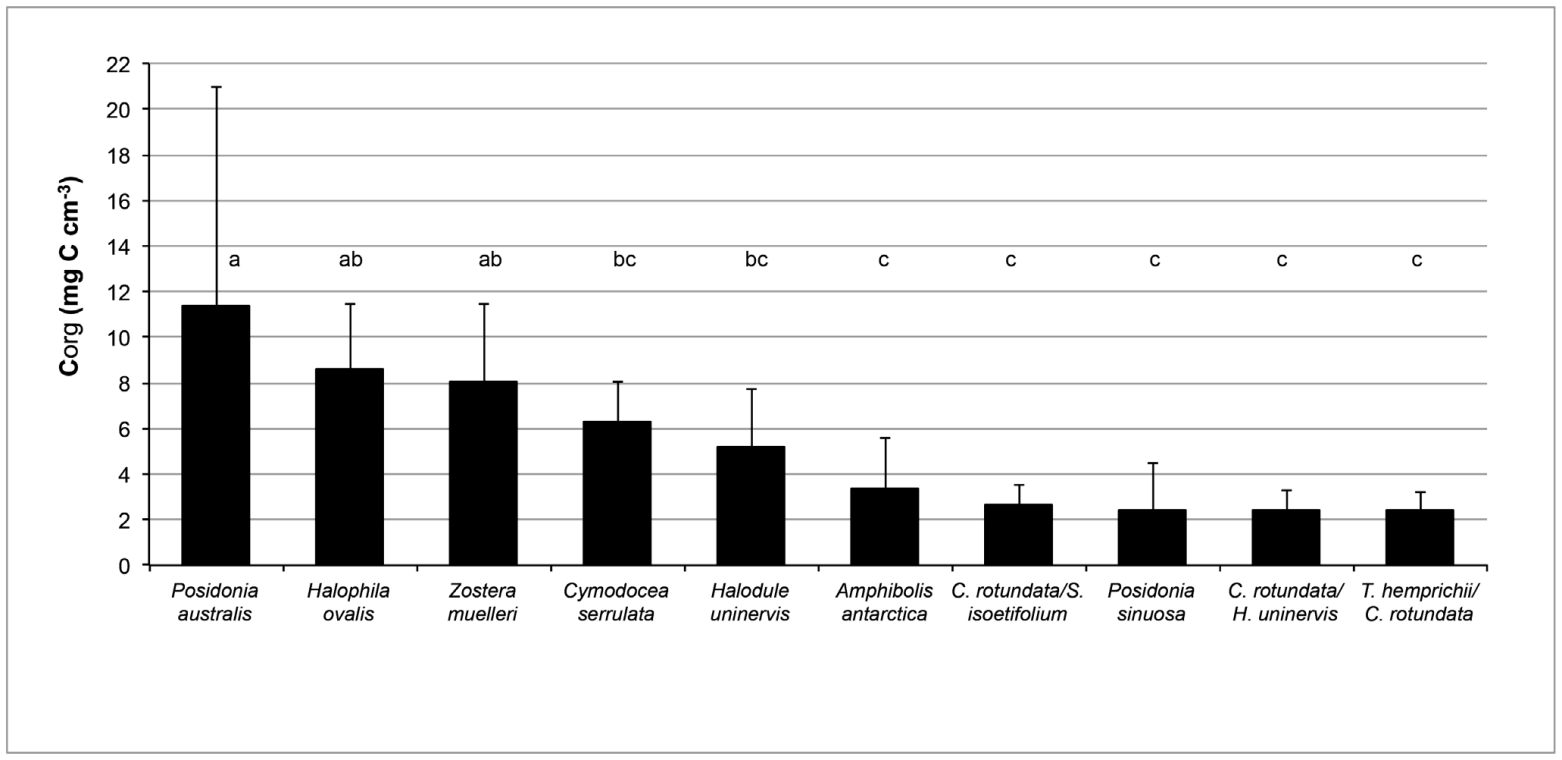
Organic carbon stocks (Mean ±SD) in the top 25 cm of sediment cores from different Australian seagrass meadows. Shared letters above the bars indicate no significant difference (p>0.05) among habitats.

The mean organic matter content of the sediments in the Australian seagrass habitats sampled ranged from 0.67 to 9.09% DW, with a mean of 3.74 ± 3.13% (Mean ± SD; Table 4). The bulk % C_org_ ranged from 0.1 to 2.14% DW, with a mean of 0.64 ± 0.68%. The range across all 17 sub-habitats was 1.09 ± 0.32 mg C_org_ cm^−3^, in a shallow temperate *Posidonia sinuosa* habitat to 20.15 ± 10.95 mg C_org_ cm^−3^ in a temperate, estuarine *P. australis* habitat (Table 4). When averaging over all the sub-habitats in which a species was sampled, the mean C_org_ stocks in the top 25 cm of the different seagrass habitats differed significantly (Fig. 1; ANOVA d.f = 9, 269; F = 17.87; p <0.001). *P australis* had the highest mean C_org_ stock (11.42 ± 9.55 mg C_org_ cm^−3^; Fig. 1). The mean C_org_ stocks for *Halophila ovalis*, and *Zostera muelleri* habitats were not significantly different to those of *P. australis*, while a mixed meadow of *T. hemprichii*/*C. rotundata* had the lowest stock (2.38 ± 0.85 mg C_org_ cm^−3^, though this was not significantly lower than a variety of sub-tropical and temperate habitats. Averaged over all sub-habitats mixed meadows of tropical species had the lowest stocks, generally less than 2.7 mg C_org_ cm^−3^.

**Table 4:** Sediment characteristics of Australian seagrass habitats and *Posidonia oceanica*.

Species	Climate	Habitat	N	% OM	s.d.	% C (bulk)	s.d.	mg C cm^−3^	s.d.	g C m^−2^	s.d.
***Posidonia australis***		**all**	**47**	**6.43**	**3.88**	**1.31**	**1.06**	**11.42**	**9.55**	**2741.10**	**2292.35**
	**temperate**	*subtidal*	*18*	*5.88*	*2.82*	*2.14*	*1.19*	*20.14*	*10.94*	*4832.88*	*2040.08*
	**subtropical**	*all*	*29*	*6.78*	*4.43*	*0.79*	*0.50*	*6.01*	*1.98*	*1442.75*	*474.36*
		*intertidal*	*14*	*4.31*	*1.81*	*0.55*	*0.19*	*4.92*	*0.89*	*1179.76*	*214.20*
		*subtidal*	*15*	*9.09*	*4.94*	*1.01*	*0.60*	*7.03*	*2.18*	*1688.21*	*523.18*
***Halophila ovalis***	**tropical**	**subtidal**	**15**	**6.21**	**3.45**	**1.18**	**0.38**	**8.64**	**2.86**	**2072.77**	**685.41**
***Zostera muelleri***	**tropical**	**subtidal**	**15**	**4.48**	**1.30**	**1.33**	**0.83**	**8.06**	**3.38**	**1933.85**	**810.48**
***Cymodocea serrulata***	**tropical**	**subtidal**	**15**	**3.02**	**0.88**	**0.68**	**0.19**	**6.32**	**1.74**	**1516.70**	**417.02**
***Halodule uninervis***	**tropical**	**all**	**27**	**5.87**	**2.51**	**0.69**	**0.36**	**5.19**	**2.55**	**1244.96**	**610.93**
		*intertidal*	*13*	*4.66*	*2.05*	*0.62*	*0.48*	*5.50*	*3.36*	*1319.62*	*805.92*
		*subtidal*	*14*	*7.00*	*2.42*	*0.75*	*0.21*	*4.90*	*1.54*	*1175.63*	*369.02*
***Amphibolis antarctica***		**all**	**59**	**2.43**	**2.41**	**0.36**	**0.32**	**3.33**	**2.26**	**799.34**	**543.57**
	**subtropical**	*intertidal*	*15*	*2.47*	*1.15*	*0.25*	*0.10*	*2.80*	*1.20*	*672.28*	*288.77*
		*subtidal*	*15*	*4.16*	*3.56*	*0.54*	*0.28*	*4.84*	*1.60*	*1162.70*	*384.96*
		*all*	*30*	*3.32*	*2.74*	*0.39*	*0.26*	*3.82*	*1.74*	*917.49*	*417.14*
	**temperate**	*subtidal 5m*	*15*	*0.79*	*0.73*	*0.13*	*0.05*	*1.54*	*0.45*	*369.01*	*108.49*
		*subtidal 10m*	*14*	*2.29*	*1.90*	*0.55*	*0.45*	*4.20*	*3.29*	*1007.23*	*790.54*
		*all*	*29*	*1.51*	*1.59*	*0.33*	*0.38*	*2.82*	*2.64*	*677.12*	*633.55*
***C. rotundata/ S. isoetifolium***	**tropical**	subtidal	15	3.08	0.39	0.32	0.11	2.67	0.85	640.15	204.87
***Posidonia sinuosa***	**temperate**	**all**	**43**	**1.72**	**1.70**	**0.28**	**0.31**	**2.44**	**2.01**	**585.35**	**482.62**
		*subtidal 5m*	*15*	*0.67*	*0.24*	*0.10*	*0.04*	*1.09*	*0.32*	*261.93*	*75.81*
		*subtidal 10m*	*28*	*2.26*	*1.87*	*0.39*	*0.35*	*3.16*	*2.17*	*758.61*	*519.71*
***C. rotundata/H. uninervis***	**tropical**	**all**	**28**	**2.55**	**2.89**	**0.28**	**0.10**	**2.43**	**0.85**	**582.33**	**203.38**
		*intertidal*	*13*	*3.74*	*3.80*	*0.28*	*0.10*	*2.28*	*0.73*	*546.42*	*174.33*
		*subtidal*	*15*	*1.51*	*1.12*	*0.28*	*0.10*	*2.56*	*0.95*	*613.45*	*226.91*
***T. hemprichii/C. rotundata***	**tropical**	**intertidal**	**15**	**2.94**	**2.36**	**0.30**	**0.10**	**2.38**	**0.85**	**571.82**	**204.72**
**All Species (Australia)**			**280**	**3.74**	**3.13**	**0.64**	**0.68**	**5.26**	**5.46**	**1262.05**	**1483.37**
*Posidonia oceanica*			**7**	**42.99**	12.09	**17.85**	6.08	**20.16**	9.49	**4837.42**	2276.62
*P. oceanica*/Australian Spp				11×		28×		4×		4×	

Data are means for the top 25 cm of sediment. s.d. = standard deviation. %C is for the bulk sample. Bold indicates mean of all habitats for a species; italics relates to individual habitats for a given species.

Several species of seagrass were sampled in more than one sub-habitat and generally showed significant variability in C_org_ stocks among sub-habitats. Thus, while a temperate *P. australis* meadow had the highest absolute stock recorded in any single sub-habitat (20.15± 10.95 mg C_org_ cm^−3^ ), a sub-tropical meadow in Shark Bay had a relatively low stock of 4.92 ± 0.89 mg C_org_ cm^−3^. Similarly, among *P. sinuosa* the C_org_ stock ranged from 1.09 ± 0.32 mg C_org_ cm^−3^, in a shallow temperate meadow, the absolute lowest value recorded in any of the sampled sub-habitats, to 3.16 ± 2.17 mg C_org_ cm^−3^ in a deeper temperate meadow.

Integrated over the depth profile of 24 cm that was sampled, the C_org_ content of the upper meadows ranged from 262 ± 75.8 g C_org_ m^−2^ for the temperate *Posidonia sinuosa* meadow to 4833 ± 2040 g C_org_ m^−2^ for temperate, estuarine *P. australis* meadow.(Table 4).

While there were significant differences in the total C_org_ stock of the different meadows, these did not consistently fall into a temperate-tropical divide; while the smallest stocks were found in a tropical species, tropical *H. ovalis* had the second largest stock. Post-hoc pairwise comparisons (Fig. 1) indicated that highest stocks were found in the temperate *Posidonia australis* and the tropical *H. ovalis*, *Zostera muelleri* and *Cymodocea serrulata* meadows, while the lowest stocks were found in a mixture of tropical, sub-tropical and temperate meadows (*Amphibolis antarctica*, *P. sinuosa* and mixed meadows of *C. rotundata* with other species).

**Figure 2:**
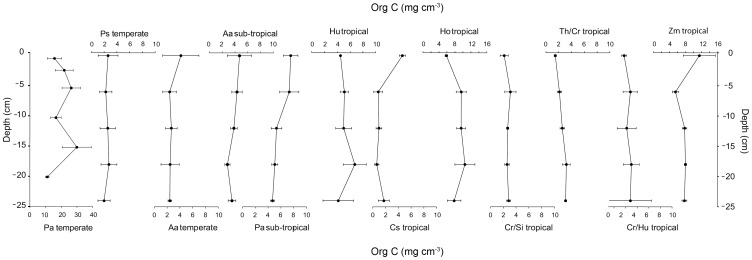
Profiles of organic carbon stocks in the top 25 cm of cores from different Australian seagrass meadows. All data are means ± std error.

The profiles of C_org_ stocks through the top 25 cm of the sediment cores also showed no consistent difference among climatic zones (Fig. 2). Temperate and sub-tropical meadows showed a general pattern of declining C_org_ stocks with depth. While some tropical meadows showed the opposite trend (increasing C_org_ stocks with depth in *Halophila ovalis* and meadows of *Cymodocea rotundata* mixed with *Thalassia hemprichii*, S*yringodium isoetifolium* or *Halodule uninervis*), others showed the same trend as the temperate meadows, of declining stocks with depth.

**Figure 3:**
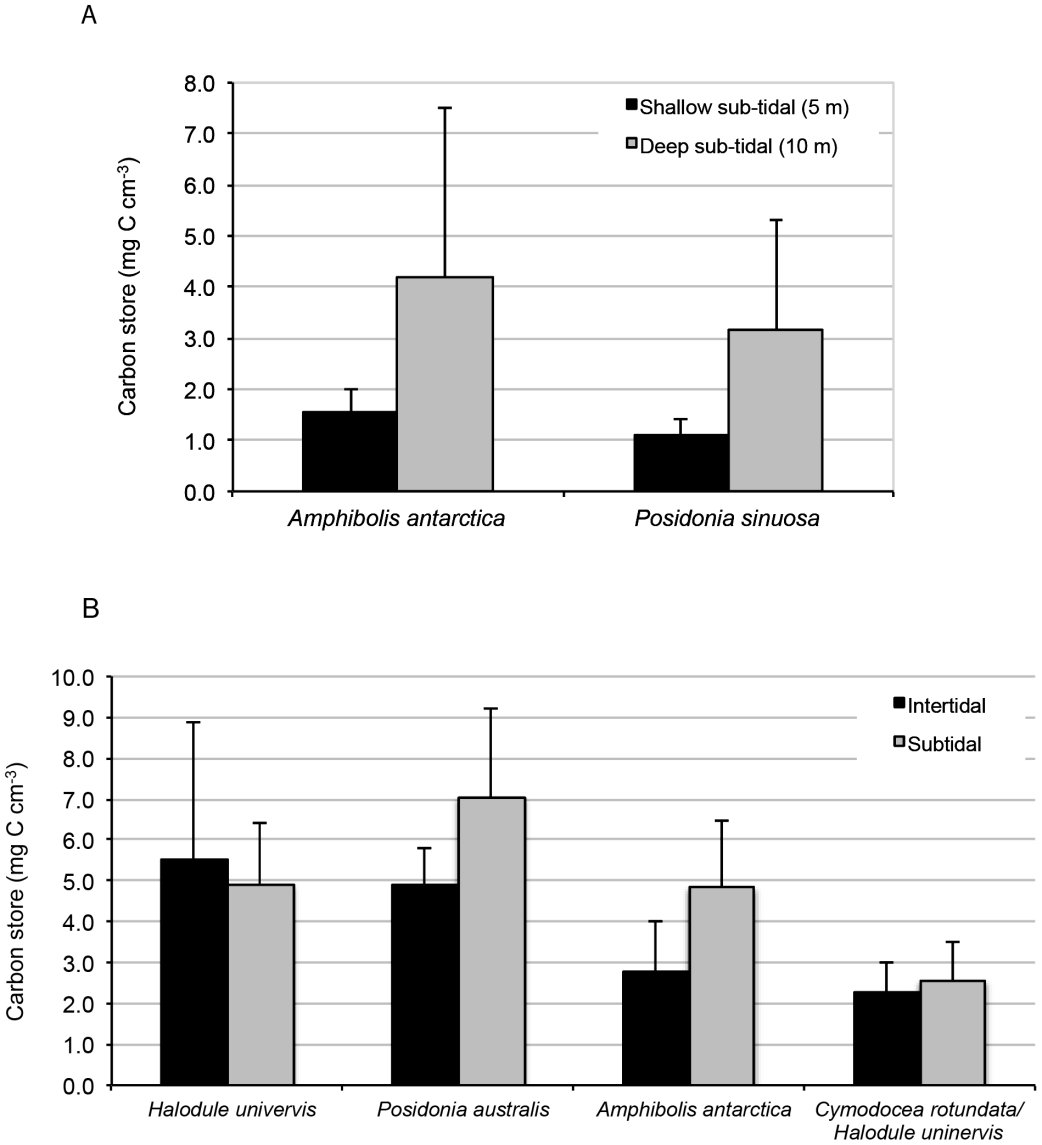
Carbon stocks in sediments of seagrass meadows occurring at different water depths: A) comparison of sub-tidal habitats (5 m vs 10 m depth); and B) comparison of inter-tidal and sub-tidal habitats.

Of those species that were sampled in more than one habitat, some showed significant among-habitat differences in C_org_ stocks while others did not. For both *Posidonia sinuosa* and *Amphibolis antarctica* there was a significant effect of water depth on the C_org_ stocks (Table 5), with greater stocks in the 10 m deep sites than the 5 m deep sites (Fig. 3a). In both cases, the stock was also much more variable in the deeper sites. In contrast, for species sampled in both inter- and sub-tidal habitats, there was no habitat effect on the C_org_ stocks (Table 5), though there were differences in stocks among species (Fig. 3b).

**Table 5:** Results of statistical testing (2-way ANOVA) for significant effects of Species and Habitat (Deep v Shallow (A) or Tidal v Inter-tidal (B)) on the C_org_ storage in the top 25 cm of seagrass sediments.

A) Deep v Shallow sub-tidal meadows of P. sinuosa and A. antarctica					
Effect	SS	d.f.	MS	F	p
Species	9.13	1	9.13	2.28	0.135
Depth	92.98	1	92.98	23.25	<0.001
Spp x D	1.45	1	1.45	0.36	0.549
Error	271.91	68	3.99		
Pairwise (Tukeys HSD) comparisons on significant effect: depth					
*A. antarctica*	Deep v shallow		p = 0.003		
*P. sinuosa*	Deep v shallow		p = 0.010		
***B) Inter-tidal v Sub-tidal meadows of four different seagrass species***					
Species	252.5	1	2159.5	526.6	<0.001
Tidal regime	12.1	1	12.1	2.9	0.088
Spp x Tide	27.1	3	9.0	2.2	0.090
Error	271.91	68	3.99		

### Estimates of Australian Seagrass Sedimentary Carbon Stocks & Accumulation Rates

The estimated C_org_ stock of the top 25 cm of sediment in Australian seagrass habitats that took into account inter-habitat variability was in the order of 155 Mt (Table 6). The majority of this stock (54%) was in the temperate seagrass habitats, dominated by the larger, meadow-forming species of *Posidonia* and *Amphibolis*. The remaining 46% was in tropical seagrass habitats of northern Australia, which are dominated by a variety of smaller-sized seagrasses that typically have a lower sedimentary C_org_ stock than the larger temperate species but a larger reported areal coverage. The estimates of annual C_org_ accumulation for Australia ranged from 0.093 Mt when applying a sedimentation rate of 0.15 mm y^−1^ to 6.157 Mt at 9.9 mm y^−1^ (Table 7). At a sedimentation rate of 1.5 mm y^−1^, which, on limited dating evidence, we believe is more representative of accumulation rates in seagrass ecosystems, the annual organic carbon accumulation rate was 0.932 Mt.

**Table 6:** Carbon storage in the top 25 cm of Australian seagrass ecosystems and those that would be estimated by applying the carbon storage values of *P. oceanica* to the same area of seagrass.

Region	Area	Habitat	C_org_ stock	C_org_ stock
	(km^2^)		(mg cm^−3^)	(Mt)
NSW	161	Zm/Pa	11.74	0.472
TAS	845	Pa/Ps/Aa(Temp.)	8.47	1.788
VIC	470	Pa(subtidal)/Aa(Temp.)	11.48	1.349
SA	9620	Pa, Ps, Aa,	8.47	20.361
QLD/NT/TS	56473	Ho, Zm, Cs, Hu, Cr/Si, Cr/Hu, Th/Cr	5.10	71.957
WA	25000	Pa, Ps, Aa,	9.53	59.558
Total*	**92569**			**155.487**
*% emissions*				*104*
***P. oceanica***	92569	Po	20.16	**466.545**
*% emissions*				*313*

C_org_ stock is the product of the area of seagrass (see Table 6) and the mean carbon storage (Table 2) of the seagrasses most likely to dominate those areas. % emission refers to the sedimentary C_org_ in Australian seagrass ecosystems as a % of annual CO_2_ carbon emissions in Australia. (Zm = *Zostera muelleri*; Pa = *Posidonia australis*; Ps = *Posidonia sinuosa*; Aa = *Amphibolis antarctica*; Ho = *Halophila ovalis*; Cs = *Cymodocea serrulata*; Hu = *Halodule uninervis*; Cr = *Cymodocea rotundata*; Si = *Syringodium isoetifolium*; Th = *Thalassia hemprichii*; Po = *Posidonia oceanica*).

**Table 7:** Estimated annual carbon accumulation rates of Australia’s seagrass habitats.

Region	Area (km^2^)	Storage (km^2^)	Annual C_org_ accumulation at different sediment accumulation rates			
			(Mt y^−1^)			
			0.15 mm y^−1^	1.5 mm y^−1^	9.9 mm y^−1^	
NSW	161	11.74	<0.001	0.002	0.019	
TAS	845	8.47	0.001	0.011	0.071	
VIC	470	11.48	0.001	0.008	0.053	
SA	9620	8.47	0.012	0.122	0.806	
QLD/NT/TS	56473	5.10	0.043	0.432	2.850	
WA	25000	9.53	0.036	0.357	2.358	
**Total**	**92569**		**0.093**	**0.932**	**6.157**	
*% of annual emissions*			*0.063*	*0.625*	*4.129*	
***Mediterranean***	*92569*	***20.16***	***0.280***	***2.799***	***18.476***	
*% of annual emissions*			*0.188*	*1.877*	*12.389*	

Estimates are based on the area of seagrass in different regions of Australia (see Table 2), the mean C_org_ stocks (Table 4) of the seagrasses most likely to dominate those areas, and a range of sediment accumulation rates derived from the literature and unpublished data (Table 3).

## Discussion

### Variability in C Stocks

This spatially-limited study has demonstrated a significant variability in the C_org_ stock in sediments beneath different seagrass habitats. Among the finding is a strong indication that a variety of biotic (species) and abiotic (habitat physic-chemical conditions) exert a strong influence on the carbons tocks of seagrass meadows, producing significant inter-habitat variability.

*Posidonia australis* had the highest C_org_ stocks, both averaged over all the sub-habitats in which it was sampled and in any individual sub-habitat sampled. This is consistent with a general expectation that larger seagrasses are likely to have larger carbon stocks due factors that affect the production, form and preservation of organic carbon. Larger seagrasses, such as *P. australis*, tend to have deeper, larger, more persistent rhizomes, often characterised by more refractory forms of structural carbon, more likely to be preserved in marine sediments than simpler, more labile forms of carbon [18]. The deeper canopy of larger seagrasses may also reduce near-bottom mean water velocities [24] enhancing particle trapping [25], [26] and reducing the resuspension of particles within the canopy [27], leading to higher inputs of allocthonous sedimentary organic matter inside macrophyte beds compared to unvegetated areas [25].

Despite *P. australis* having the largest stock of organic carbon, there was considerable variability among habitats for this species. Among the three sub-habitats sampled, there was a 4-fold range in the stocks, with the inter-tidal sub-tropical habitat having least and the sub-tidal, temperate habitat the most. This indicates a strong effect of abiotic variables on the carbon storage capacity of this species. The temperate meadow was located in Waychinicup inlet, a small, relatively sheltered coastal estuary. The combination of sheltered conditions and the inputs of allocthonous carbon from its small catchment [28] may contribute to the higher carbon stock at this site. In contrast, the sub-tropical meadow was located in very shallow water (<0.1 m at spring low tide), which would enhance hydrodynamic exposure and associated resuspension and export of sedimentary carbon matter. It is also likely that the generally warmer conditions of Shark bay where the meadow was located, and the shallowness of the site would result in higher mean temperatures that would facilitate enhanced remineralisation of sedimentary carbon [29]. Elsewhere, shallow *P. oceanica* meadows were observed to have higher rates of carbon remineralisation than deeper meadows [30], attributed to higher respiratory rates at the shallow sites. The effect of depth on carbon stocks is discussed further below.

Surprisingly, *Halophila ovalis*, which has small leaves and a very short canopy and root system relative to the other species, had the second-highest mean C_org_ stock, which was not significantly different to that of *Posidonia australis* and was greater than many larger species. The relatively high C stocks in *H. ovalis* meadows may be explained by their morphology and their habitat characteristics. Despite having smaller leaves than other seagrass species, *Halophila decipiens* increased the threshold velocity for sediment motion similar to larger seagrasses [31]. The allocation of leaf biomass and rhizomes closer to the sediment-water interface when compared to other seagrasses was hypothesized as the main physical basis for the significant sediment stabilization effects proved by *H. decipiens*. It has also been observed that at some water velocities denser seagrass canopies can induce ‘skimming flow’ which directs particles over the canopy and reduces the capture efficiency [32], which may explain some of the differences between *H. ovalis* and the larger meadows. Further, a global study of seagrass sediments found that, on average, 50% of the sedimentary C_org_ matter in seagrass meadows was derived from allocthonous sources [12]. In the case of *Halophila*, it is possible that despite its low biomass the canopy is capable of trapping a variety of C sources resulting in the relatively high carbon stocks. However, it seems more probable that this species is adapted to living in naturally depositional environments and the seagrass itself is a relatively minor contributor to the stocks. This hypothesis is supported by the stable C isotopic composition of the sediments studied for *H. ovalis*. While the average δ^13^C value of the tissues of this species has been determined to be below −14 ‰ (e.g. [12], [33]), the value for the sediments analysed for *H. ovalis* averaged −24.7 ‰ (data not shown), indicating a potentially strong contribution from algal production but, more likely (given the location of the *H. ovalis* meadows sampled for this study), from terrestrial inputs. Further studies into the sources of C would help to clarify the relative importance of seagrasses in contributing to C stocks.

The data indicate that both the species of seagrass and the abiotic habitat characteristics are important in driving variability of sedimentary C_org_ stocks. While the habitats we studied were characterised by one or two dominant seagrasses, we cannot conclude that these same species were dominant at the study sites over the duration of carbon accumulation to a depth of 25 cm. However, given typical sediment accumulation rates in seagrass habitats of between 1 and 10 mm y^−1^ (Table 3), the 25 cm deep cores may represent a period in the order of 25–250 years. For some of the smaller, disturbance-adapted species, it is likely that the seagrass composition at the sites may have changed during that time, while the larger, perennial species might be expected to have dominated the sites for most, if not all, of that time, and at least some of the variability in C_org_ stocks is related to the species of seagrass.

Water depth had a significant effect on C_org_ stocks. For *Posidonia sinuosa* and *Amphibolis griffithiii,* in Geographe Bay there were larger C_org_ stocks in the deeper sites than the shallow sites. The higher net productivity of meadows at shallow sites compared to deep sites [15], [34] would suggest greater carbon inputs and sedimentary organic carbon at shallow sites. However, shallower meadows may also be exposed to greater hydrodynamic forces and export of C (as wrack) may be greater than at deeper sites which, in addition, can be receiving environments for organic matter from shallower sites [30]. This hydrodynamic exposure may also result in greater exposure to oxygenated conditions for the shallower sediments [35], promoting higher respiration rates and detrital decay than in deeper sites, as observed in *P. oceanica* meadows [30]. Furthermore, higher sediment deposition rates at deeper sites (due to lower hydrodynamic forces) may contribute to greater vertical growth rates of seagrasses and, therefore, C_org_ accumulation rates and stock. Based on our results, even very similar meadows may have significantly different C storage capacities due to a combination of factors other than species composition. Water depth in particular should be considered potentially important in affecting C_org_ stocks in the surficial sediments and warrants further study.

Surprisingly, and in contrast to sub-tidal water depth, when a species occurred in both inter-tidal and sub-tidal habitats there were no differences in sedimentary organic carbon stocks. The effects of intermittent submergence on primary production and community respiration are complex. Inter-tidal habitats are likely to experience higher temperatures and associated respiration rates which would enhance C remineralisation [18] and lower C accumulation, particularly if tidal currents also contribute to the export of particulate matter and oxygenation of surface sediment layers. On the other hand, experimental studies have shown that some species of seagrass exhibit higher rates of photosynthesis (ETR) in air than when submerged [36]. In addition, the rates of plant respiration can be lower in inter-tidal sediments [37], and gross community metabolism is reduced during emersion periods [38], which may promote C_org_ preservation through reduced rates of remineralisation. A complex set of factors seems to be interacting as systems shift between intertidal and subtidal states.

As our study was not orthogonal in its design, it is not possible to draw definitive conclusions regarding the difference that climatic region may make to C_org_ stocks. However, as with inter-tidal and sub-tidal habitats, there was no consistent difference in the C storage of tropical, sub-tropical and temperate seagrass habitats. It is generally thought that the higher temperatures in tropical regions promote more efficient remineralisation of soil organic matter, and a similar process may well occur in shallow coastal sediments. However, while this simple models may hold for labile forms of C the refractory nature of the C substrate and a range of physico-chemical process that protect C from remineralisation may result in poor correlations between temperature and total C stocks in soils [39]. Given the high proportion of complex forms of C in seagrass rhizome material [40], [41], their relative resistance to microbial degradation [29] and the likelihood of oxygen exclusion in deeper sediments, it is likely that similar complex degradation processes relate to seagrass sedimentary C_org_ and that simple tropical-temperate divisions based on temperature are unlikely to be the main drivers.

The vertical profile of carbon accumulation varied among the species, with most showing the expected decrease in organic matter with depth and other showing the inverse. The decrease with depth is typical of sedimentary systems where there is little turnover of the sediment profile and carbon diagenesis results in a gradual loss of first labile and then increasingly refractory C [18]. We did not examine the cores for faunal biomass, sediment grain size or sediment dating, which may provide insights into the degree of mixing of surface sediments, but these processes may contribute to the observed differences in profiles for those cores that did not show a decline with depth. Also, it is probable that the top 25 cm is not sufficiently deep to describe the expected negative exponential profile of organic matter decomposition with aging (e.g. *P. oceanica* [42]).

### Implications of Variability among Seagrass Habitats for Regional Estimates of Carbon Accumulation

While our data are limited in their spatial coverage and in the degree of habitat replication, the18-fold difference in the C_org_ storage among the different seagrass sediments is clear evidence of the significant variation among seagrass habitats. The C_org_ storage values presented are likely to be significant under-estimates of the true storage on an areal basis as they only apply to the top 25 cm of sediment. Many seagrass meadows have organic-rich sediment extended deeper than this, especially species of *Posidonia*, with mats cored to depths of over 2.5 m depth [23], [42], [43]. Furthermore, the mapping of seagrass area is incomplete for much of the country, including large regions such as the NW, and so the total area of seagrass is also likely to be an underestimate. Conservatively, it is reasonable to assume the storage is at least double that which we have estimated. If we accept our estimated stock of 155 Mt (areal stock: 1.61×10^9^ g C_org_ km^−2^), this equates to approximately 100% of the country’s annual CO_2_ emissions [44] stored in the top 25 cm of sediments beneath Australian seagrass meadows, a significant portion when considering that Australia is one of the most intensive per capita greenhouse gas emitters in the World. This stock represents 7.7–15.3% of the recent global estimate for the top meter of world seagrass soils [21]. The average areal stock estimated by these authors for all seagrasses studied was of 19.42×10^9^ g C_org_ km^−2^, about 3 times higher than that estimated in the present study for Australian seagrasses (after roughly normalizing for the top meter of sediment). This proportion increases to 4 times when comparing the Australian average against the South Australia region considered by Fourqurean et al. [21] (26.83×10^9^ g C_org_ km^−2^), but declines to 2.3 times when comparing the average for South Australia in our study with their South Australian estimate. Again, these wide ranges and discrepancies between studies highlight the need to augment our knowledge about the natural variability of organic carbon stocks in seagrass soils.

Our estimates of the annual accumulation of C_org_ by Australian seagrass ecosystems was in the order of 0.09–6.15 Mt C_org_. y^−1^ (1.01–66.5 t km^−2^ y^−1^). As explained previously, we consider a sediment accumulation rate of 1.5 mm y^−1^ to be more typical for seagrass ecosystems than the lower and upper estimates, and this sediment accumulation rate yields an estimated C_org_ accumulation rate of 0.93 Mt y^−1^, or 10.06 t km^−2^ y^−1^. At this rate, Australian seagrass ecosystems would be sequestering up to 0.6% of the country’s estimated annual CO_2_ emissions (Australian Government Geoscience Australia; www.ga.gov.au). These estimates of C accumulation by Australian seagrass ecosystems are significantly lower than previously published estimates for seagrasses but still significantly higher than those reported for most of the World’s ecosystems (Table 8). This supports earlier assertions [2], [11] that seagrasses have a high carbon sequestration potential, on a per unit area basis. The Australian Government has set the price of carbon at A$25.40 in 2014–15, after which it will be determined in the market, with estimated trading price of $35 t^−1^ in 2020 [45]. Using the estimated national seagrass C_org_ stock of 155 Mt and the above pricing, seagrass habitat would have a market value of $3.9–5.4 bill. for its carbon sequestering function alone. While he free-market trading price of carbon will vary from this predicted value, these estimates serve to emphasise the value of seagrasses and the need to conserve and restore these ecosystems.

**Table 8:** Estimated annual C_org_ stocks in soils of different ecosystems.

Ecosystem	Soil/sediment Carbon Accumulation rate
	(g C m^−2^ y^−1^)
Tropical Forests	2.3–2.5
Temp Forests	1.4–12
Boreal Forests	0.8–2.2
Temp grassland	2.2
Temperate desert	0.8
Tundra	0.2–0.7
*Posidonia oceanica*	*6–175*
***Australian seagrasses***	***1.0–66.5*** *
	**Soil/sediment Carbon Stock**
	**(g C m^−2^)**
Tropical montane	6100
Tropical wet	6100
Tropical moist	5000
Tropical dry	2200
Warm Temperate moist	7600
Warm temperate dry	2600
Cool temperate moist	11600
Cool temperate dry	4900
Boreal	14900
Polar	11800
***Australian seagrasses***	***1090 – 20140^#^***
*P. oceanica*	10500–40000^∧^

Data from [67] except Australian seagrasses (this study) and *P. oceanica* (based on the range reported [5], [7], [42], [47].

* ^∧^Value assume sediment depth of 1 m, which is a conservative estimate for the higher values which are for *Posidonia* ecosystems with reported organic sediment depths of >2.5 m ([23] and Pers. Obs. for Oyster Harbour and Waychinicup Inlet in SW Australia; P. oceanica references as above).

### Comparison with *P. oceanica*-based Estimates

The mean C_org_ content of Australian seagrass habitats was about 4 times lower than that recorded in *P. oceanica* meadows from the Mediterranean Sea. However, the highest C_org_ content of any Australian seagrass habitat (estuarine *P. australis* – 4833 g C_org_ m^−2^) was almost identical to that of *P. oceanica* meadow in the Mediterranean Sea (4837 g C_org_ m^−2^), when normalized to the top 25 cm. The similarity of the *P. australis* and *P. oceanica* values is consistent with their similar morphology and meadow structure. Both species have large, persistent rhizomes, placing them close to each other in seagrass functional-form models [8], [14], which accumulate in the sediments producing deep, organic-rich sediment profiles. Importantly, the meadows of *P. oceanica* reported in the literature tend to have much deeper organic sediment profiles than *P. australis* (reportedly up to 8 m compared with 2.5 m for *P. australis*) due to the vertical growth form of the rhizome. Consequently, *P. oceanica* habitat will still likely contain much larger masses of sedimentary C_org_ than any of the Australian seagrass habitats we sampled, and probably represents a global maximum among seagrasses.

Until now, most of the global estimates of seagrass carbon storage have, necessarily, been based on indirect estimates and a few direct measurements of sedimentary C_org_ stocks in *Posidonia oceanica* meadows, plus a single study of *Cymodocea nodosa* (e.g. Duarte et al. 2005, Nellemann et al. 2009). The variation in C_org_ storage values among seagrass ecosystems reported here suggests that it is important to incorporate this variability into regional or global estimates of seagrass C stocks. The effect of this inter-habitat variation is illustrated by re-calculating the C stocks and accumulation rates for the estimated area of Australian seagrass using a uniform C_org_ storage value. We applied the value reported for *P. oceanica* (20.16 mg cm^−3^; derived from the data reported in Mateo et al. (1997) and Serrano et al. (2012), since this species has typically been used as the representative seagrass in global C accumulation exercises (note: while several studies report C accumulation rates for *P. oceanica* [3], [4], [46], they ultimately rely on earlier C stock estimates [30], [47], which are applied here.

When inter-habitat variation in seagrass organic carbon stocks was ignored and we applied only the *P. oceanica* carbon storage values, the stock was estimated to be about 448 Mt and the annual accumulation 0.28–18.4 Mt y^−1^, 3-times the estimates we made using the range of carbon storage values we measured for the Australian seagrass habitats and more than 300% of Australia’s annual CO_2_ emissions (compared with 100% for the estimates incorporating inter-habitat variability). These simple comparisons effectively demonstrate the need to better document inter-habitat variability in seagrass carbon storage and to incorporate this variability into regional and global estimates of seagrass carbon stocks. The significant point is not the absolute values of carbon storage, but the significant effect on the estimate of incorporating inter-habitat variability. A 3-fold discrepancy in estimates is sufficiently large to undermine confidence in Blue Carbon estimates among economic and political decision-makers. This supports Nelleman et al.’s [2] identified need to incorporate inter-habitat variability into global estimates if the uncertainties in these estimates are to be reduced.

## Conclusions

There is considerable spatial variability in the C_org_ stock of seagrass sediments. This variability may be related to both the species of seagrass and the habitat setting in which they occur, particularly water depth. The data set presented here is limited and the errors associated with the estimates are likely to be significant, though no more so than the global estimates of seagrass C capture extrapolated from a much more limited set from the Mediterranean Sea, acknowledging that at the time those were the best available data. However, our data serve to emphasise the need for robust data sets on the carbon storage and accumulation rates in different seagrass ecosystems. There is also a pressing need to better understand the habitat features that drive this variability in C storage and accumulation rates. Assuming a uniform ability to capture and sequester carbon among the 70+ species of seagrasses will potentially lead to erroneous estimates of global C storage and improving our understanding of the variability in C stocks and accumulation rates is critical if we are to produce robust estimates of regional and global C capture and storage in seagrass ecosystems.
